# Genetic Modification of *Mucor circinelloides* to Construct Stearidonic Acid Producing Cell Factory

**DOI:** 10.3390/ijms20071683

**Published:** 2019-04-04

**Authors:** Md. Ahsanul Kabir Khan, Junhuan Yang, Syed Ammar Hussain, Huaiyuan Zhang, Victoriano Garre, Yuanda Song

**Affiliations:** 1 Colin Ratledge Center for Microbial Lipids, School of Agricultural Engineering and Food Science, Shandong University of Technology, Zibo 255000, Shandong, China; kabir_khan@sdut.edu.cn (M.A.K.K.); judywoniu@163.com (J.Y.); ammarshah88@yahoo.com (S.A.H.); zhyuan004@126.com (H.Z.); 2 Departmento de Genética y Microbiología (Unidad Asociada al Instituto de Química Física Rocasolano, Consejo Superior de Investigaciones Científicas), Facultad de Biología, Universidad de Murcia, Murcia 30100, Spain

**Keywords:** delta-15 desaturase, SDA production, homologous overexpression, *Mucor circinelloides*

## Abstract

Stearidonic acid (SDA; 18:4, n-3) is the delta 15-desaturase product of gamma linolenic acid (GLA; 18:3, n-6) and delta 6-desaturase product of alpha linolenic acid (ALA; 18:3, n-3). Construction of engineered oleaginous microbes have been attracting significant interest in producing SDA because of its nutritional value and pharmaceutical applications. *Mucor circinelloides* is a GLA producing filamentous fungus, which can be a useful tool to produce SDA. This study has, therefore, overexpressed the *delta-15 desaturase* (*D15D*) gene from *Mortierella alpina* in this fungus to construct a SDA-producing cell factory. To produce SDA in *M. circinelloides*, the homologous overexpression of *D15D* gene was analyzed. When the gene was overexpressed in *M. circinelloides* CBS 277.49, up to 5.0% SDA was accumulated in this strain. According to current knowledge, this is the first study describing the construction of a SDA-producing cell factory by overexpression of *D15D* gene in oleaginous fungus *M. circinelloides*. A new scope for further research has been established by this work to improve SDA production in this fungus, specifically in its high lipid-producing strain, WJ11.

## 1. Introduction

Polyunsaturated fatty acids (PUFAs) have significant functions in maintaining human health [1]. They are very important nutritionally because mammals, including humans, can synthesize saturated fatty acids (SAFAs) and mono-unsaturated fatty acids (MUFAs) but cannot produce linoleic acid (LA; 18:2, n-6) and α-linolenic acid (ALA; 18:3, n-3) that are known as de novo omega-6 or omega-3 polyunsaturated fatty acids (PUFAs), respectively. Hence, these fatty acids are called essential fatty acids (EFAs) and must be obtained from external sources [1,2]. LA and ALA are metabolized to arachidonic acid (ARA; 20:4, n-6) and eicosapentaenoic acid (EPA; 20:5, n-3), respectively, in a series of reactions catalyzed by the same sets of enzymes [3]. However, the metabolism of EFAs can be altered in several diseases, such as hypertension, diabetes mellitus, obesity, atherosclerosis, Alzheimer’s disease, schizophrenia, cancer, etc. Therefore, the physiological role of EFAs and their products is very important as they are involved in various biological reactions [4]. Hence, PUFAs are attracting significant research interest in recent times [3].

**Figure 1:**
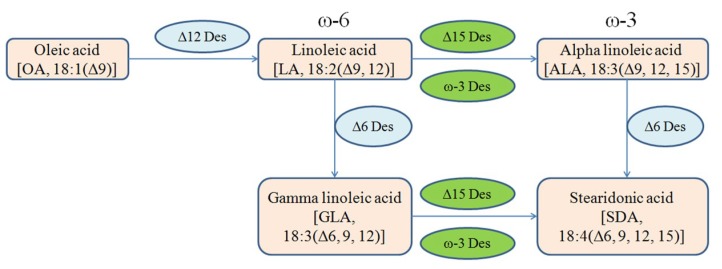
Metabolic pathway of linoleic acid (LA) flux into n-6 or n-3 PUFAs to produce stearidonic acid (SDA).

Stearidonic acid (SDA; 18:4, n-3) is produced from gamma linolenic acid (GLA; 18:3, n-6) and ALA (18:3, n-3) catalyzed by the enzymes delta 15-desaturase and delta 6-desaturase, respectively (Figure 1) [4]. SDA can be elongated to a longer chain omega-3 PUFA with related biological properties, such as EPA and docosahexaenoic acid (DHA) [5]. However, conversion of SDA from ALA is very inefficient in humans because delta 6-desaturase is rate-limiting, therefore it is difficult to change tissue levels of EPA (20:5, n-3) by the consumption of ALA. Consequently, the oral administration of SDA has been suggested as an alternative approach of increasing the amount of EPA in body tissues [6,7]. Again, in comparison to EPA and DHA, SDA is less unsaturated, more stable, less prone to oxidation, and therefore more amenable to a wide variety of food and beverage applications [8].

SDA occurs naturally in seafood, fish and a few species of seaweed as a minor n-3 PUFA [9]. Among the plant sources, oils derived from blackcurrant seed and members of the *Boraginaceae* family contain SDA [10]. By weight, Echium oil is comparatively a better plant source of SDA and contains both ω-3 PUFA and ω-6 PUFA [11]. However, due to safety concerns, limited shelf-life, palatability issues and the risk of over-fishing, fish and fish oil are difficult to use as commercial source of (ω-3) LCPUFA [12,13]. Plant sources also need to be improved for large scale production due to their low yield. Therefore, there is a requirement to identify alternative sources of SDA that can be used commercially [14].

Fungi, microalgae and bacteria that can accumulate lipids up to 20% of their dry cell weight are known as oleaginous microorganisms. *Mucor circinelloides* has been extensively investigated for GLA production since the 1980s [15]. It has been considered as an important classical organism for microbial lipid analysis because of its ability to produce oil that is rich in GLA as well as the availability of genome data and genetic tools [16]. In previous studies this fungus was used for improved GLA production [15], increased lipid accumulation [17], 13C-metabolic flux analysis of lipid accumulation [18], investigation of the effects of 20 standard amino acids on growth, GLA and total fatty acids production [19], the role of pentose phosphate pathway in lipid accumulation [20], etc. *Mortierella alpina,* another filamentous fungus, can accumulate lipids up to 50% of its dry weight [21,22] and mostly composed of triacylglycerol with a high amount of arachidonic acid (AA; 20:4, n-6) [23,24], however at a low temperature it can also produce EPA [25]. In this experiment, the *delta-15 desaturase (D15D)* gene from *M. alpina* was cloned and recombined in *M. circinelloides* to make a SDA-producing cell factory.

## 2. Results

### 2.1. Generation of D15D-Overexpressing Strains of *M. circinelloides* by Genetic Engineering

According to the genomic data of *M. alpina*, this study found only one gene encoding for D15D (Genebank accession number KF433065), which is 1212 bp long. For overexpression of the target gene, the coding region of *D15D* was cloned into the *M. circinelloides* expression vector pMAT1552, which contained the strong *zrt1* promoter and flanking sequence of *carRP* locus to allow integration of the whole over-expressing construction by homologous recombination [26]. Integration in *carRP* locus produced white colonies, which were easily distinguishable from yellow transformants that did not integrate the *D15D* gene. The target gene-overexpressing plasmids, pMAT1552-D15D, and the empty plasmids pMAT1552 were transformed into the uridine auxotrophic strain, pleu-MU402, and selection of the colonies was performed as reported by Rodríguez-Frómeta [27]. Three overexpressing and one control transformants named as Mc-D15D, Mc-D15D-1, Mc-D15D-2 and Mc-1552, respectively, were selected. Additional screening was carried out (data not shown) and only one strain (Mc-D15D) that produced a maximum amount of lipid and SDA was selected for further experiments.

**Figure 2:**
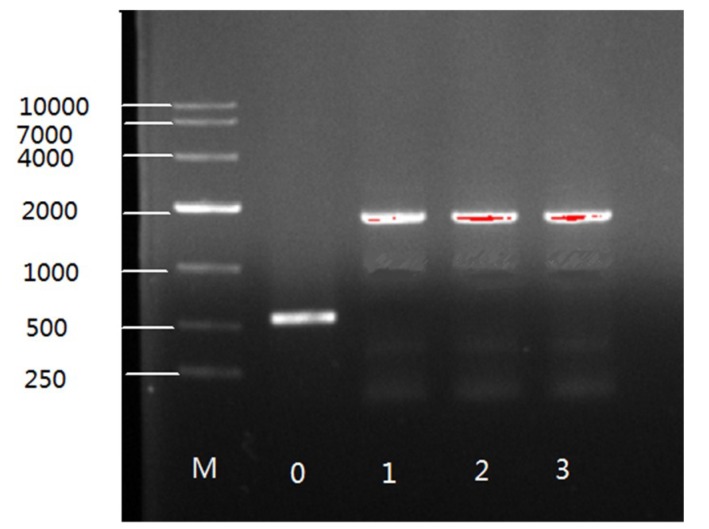
PCR amplification of genome of control and recombinant strains. Band 0 representing the control strain and 1,2,3 showing the recombinant strains.

PCR analysis was used to confirm the integration of the target gene in the genome of overexpressing transformants. A primer pair 1552-F/R (Table S1) was used to amplify the target gene and the 557 bp sequences of the plasmid pMAT1552. As expected, bands of 1796 bp of PCR products were seen on gels for transformants with the target gene but only 557 bp fragment was observed for Mc-1552 control strain (Figure 2). Thus, PCR amplification results confirmed the integration of the target gene in the genome of recombinant fungi.

### 2.2. Expression Levels of D15D Genes in the Recombinant Strains

**Figure 3:**
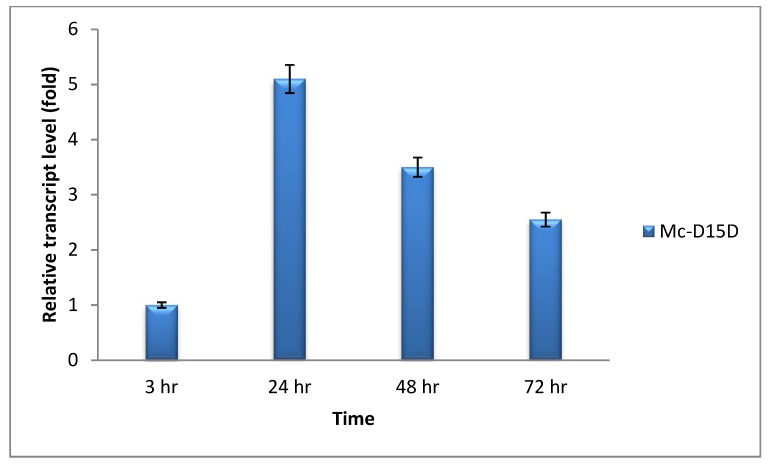
Determination of expression levels of delta-15 desaturase (*D15D*) genes by RT-qPCR in the recombinant strains.

Real-time quantitative PCR were carried out to analyze the mRNA level of *D15D* in the recombinant strains at 3, 24, 48 and 72 h of growth in two liter fermenter with K & R medium (Figure 3). The mRNA expression level of Mc-D15D was considered as 1 at 3 h and by comparing with this value, the expression level increased in that strain by 5.10, 3.50 and 2.55 fold at 24, 48 and 72 h, respectively. Although it increased quickly from 3 to 24 h, there was a decreasing trend with the incubation time after 24 h. The fact that D15D mRNA was maintained at elevated levels throughout the culture time confirmed that it was overexpressed in the recombinant strains.

### 2.3. Effect of D15D Over-Expression in Cell Growth and Lipid Accumulation

**Figure 4:**
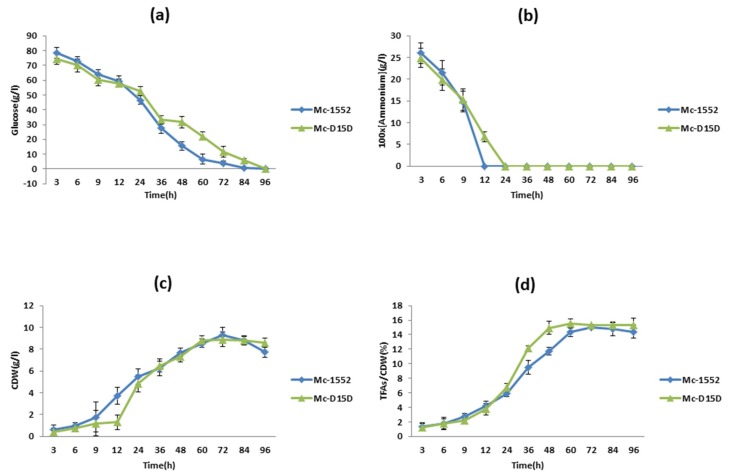
Cell growth and lipid accumulation of *D15D* overexpressing strains. Recombinant Mc-D15D and control strain Mc-1552 cultures were grown in 1.5 L modified K&R medium and (**a**) glucose concentration, (**b**) ammonium concentration, (**c**) cell dry weight (CDW), and (**d**) lipid content were measured. Samples from the fermenter were taken at the indicated times. The values were mean of three biological replicates. Error bars represent the standard error of the mean.

The effect of the *D15D* over-expression in growth and lipid accumulation was analyzed in the strain Mc-D15D, as shown in Figure 4. The concentrations of ammonium and glucose in the culture medium are also highlighted in Figure 4. Adequate concentrations of glucose remained during the entire bioprocess (Figure 4a), however at approximately 24 h the ammonium was used up by Mc-D15D (Figure 4b). After 12 h of cultivation, cell dry weight (CDW) increased rapidly but slowed down after nitrogen depletion (Figure 4c). The fungus started to accumulate lipids immediately after nitrogen exhaustion from the culture medium. The total fatty acids (TFAs) content increased rapidly from 24 h, reaching its peak at 60 h and then slowed gradually. The maximum content of TFAs was found to be 15.53% in Mc-D15D (Figure 4d).

### 2.4. SDA Accumulation in D15D Overexpressing Strains

The fatty acid composition in *D15D* genes overexpressing strains are presented in Table 1. In the transformants, SDA production started at 24 h, and at 48 h its content reached its peak (5.09%). From 36 to 72 h the SDA content remained elevated over 4%. In these recombinant fungi the other fatty acid contents were almost similar as found in the control strains.

## 3. Discussion

The accumulation of very-long-chain polyunsaturated fatty acid (VLCPUFA) in recombinant microorganisms is a process which can further improve the accumulation of desired products [28]. The oleaginous fungus, *M. circinelloides*, is an attractive oleaginous fungus for researchers because of its ability to produce oil that is rich in gamma linolenic acid. On the other hand, *M. alpina* produces both ARA and EPA and possess both ω-6 and ω-3 biosynthetic pathways for the production of fatty acids. However, in all other features, both fungi share similar characteristics and phenotype (filamentous) [1]. Delta-15 desaturase (ω-3 desaturase) of *M. alpina* ATCC 32221, is reported to convert GLA to SDA and LA to ALA. ALA is converted to SDA by delta-6 desaturase, which is already present in *M. circinelloides* [29]. Therefore, SDA can be produced using both pathways that were constructed in this fungus by overexpressing *D15D* gene (Figure 1). Besides the production of SDA, a significant amount of ALA was also produced in this process. The RT-qPCR results revealed that D15D mRNA remained elevated during the entire culture time of Mc-D15D and fatty acid analysis revealed the presence of SDA. These results confirmed that *D15D* was overexpressed in the recombinant strains.

Deficiency of nitrogen (N) is a general approach to start the storage of lipid in most oleaginous microorganisms. Investigation of lipid accumulation is frequently carried out by comparing the N rich phase to the N deficiency phase in the whole culture time, and the quantity of lipid enhances under N depletion [30,31,32]. The maximum concentration of TFAs is 15% in CBS 277.49 [16]. Similar trends and results were observed in the experiments of this study. Recombination and expression of *D15D* gene showed no significant effect on growth or total lipid content of this fungus.

In *M. circinelloides* the main fatty acids are 16:0, 18:0, 18:1, 18:2 (LA) and 18:3 (GLA) [15,16]. After overexpressing *D15D* gene, the same fatty acids were found as the major lipids and its expression did not affect the major lipid profile of this organism. SDA normally occurs only as a metabolic intermediate in the biosynthetic pathway of ω-3 fatty acids in higher plants or fungi and algae; it is not significantly accumulated in any organism [14]. In the recombinant Mc-D15D in this study, SDA is the end product of this pathway. Since *M. circinelloides* has been investigated extensively for GLA production since the 1980s [33], it can also be used for SDA production industrially.

Previous research has confirmed that during the last 50 years the decreased consumption of ω-3 fatty acids is thought to be responsible for the increased occurrence of some diseases, such as hypertension, obesity, atherosclerosis, CHD, metabolic syndrome X, cancer, etc. The precursor of the prostaglandins of series 3 and the leukotrienes of series 5 are also produced from EPA [34]. Fish and sea food products are the major sources of these ω-3 PUFA, however due to their poor stability and chemical safety these are not frequently used in food processing [7]. Current plant sources also need to be improved for large scale production [35]. Scientists are trying to construct engineered SDA-producing microbes because of nutritional value and increased demand. Kimura et al. constructed genetically modified *Saccharomyces cerevisiae* which can produce up to 13% SDA when the fungus is supplied with increased histidine [36,37]. The recombinant *M. circinelloides* in this study can produce almost 5% SDA without any supplement and according to our knowledge, this is the first study describing the overexpression of *D15D* gene in *M. circinelloides* to construct SDA producing cell factory. However, more research is required to use this fungus for the industrial production of SDA.

## 4. Materials and Methods

### 4.1. Plasmids, Strains and Conditions of Culture Media

*Mortierella alpina* ATCC 32222 was used as the source of delta-15 desaturase *(D15D)* gene. The uracil auxotroph strain, pleu-MU402 of *M. circinelloides* CB277.49 [38] was used as the recipient strain for recombination and overexpression of the desaturase gene. The expression vector pMAT1552 [39] was used for gene cloning. *Escherichia coli* Top 10 was used to maintain and propagate recombinant plasmids, which was grown in lysogeny broth at 37 °C. *Mortierella alpina* ATCC 32222 was grown in a 1-L flask containing Potato Dextrose Water (PDW) for 72 h for mycelia collection. The initial cultivation of the recombinant strains Mc-D15D (*D15D* overexpresssion strains) and Mc-1552 (control strains carrying the vector pMAT1552) were carried out in 1-L flasks containing 150 mL K&R medium containing 30 g/L glucose, 3.3 g/L ammonium tartrate, 7.0 g/L KH_2_PO_4_, 2.0 g/L Na_2_HPO_4_, 1.5 g/L MgSO_4_·7H_2_O, 1.5 g/L yeast extract, 0.1 g/L CaCl_2_·2H_2_O, 8 mg/L FeCl_3_·6H_2_O, 1 mg/L ZnSO_4_·7H_2_O, 0.1 mg/L CuSO_4_·5H_2_O, 0.1 mg/L Co(NO_3_)_2_·6H_2_O and 0.1 mg/L MnSO4·5H_2_O, pH 6.0 [40] for 24 h at 28 °C with shaking at 150 rpm. This initial culture was inoculated at 10% (*v*/*v*) into a 2-L fermenter (BioFlo/CelliGen115, New Brunswick Scientific, Edison, NJ, USA) where a modified K&R medium containing 80 g glucose/L was used as culture medium. The volume of medium was 1.5 L and fermenters were held at 28 °C, stirred at 700 rpm with aeration of 0.5 vvm. Automatic addition of 2 M NaOH was used to maintain the pH at 6.0.

### 4.2. Plasmids Construction

**Figure 5:**
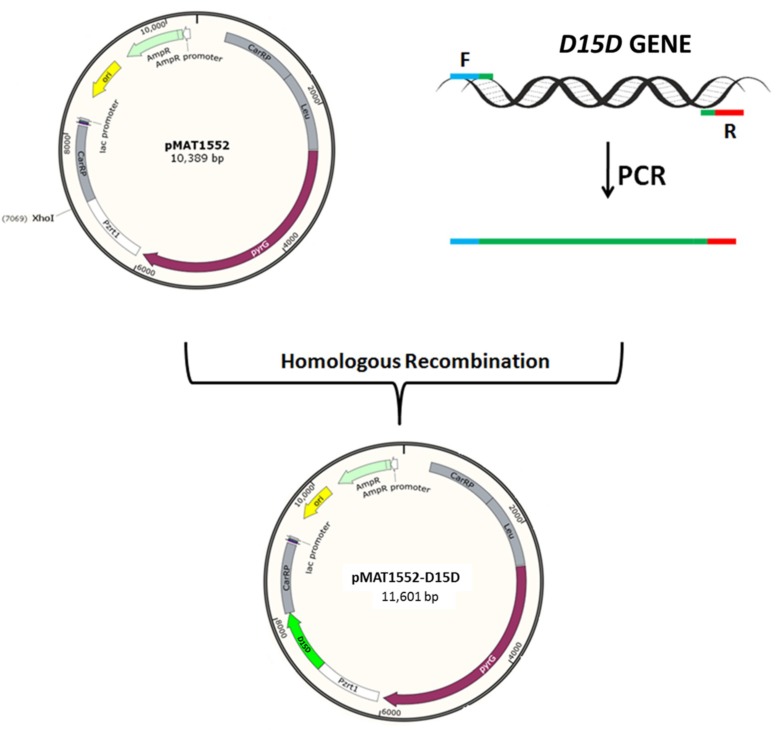
The structure of plasmids pMAT1552 and pMAT1552-D15D. The *D15D* gene was isolated by PCR amplification with appropriate primers. The PCR fragment was ligated into *XhoI* restriction site to generate plasmid as pMAT1552-D15D.

D15D-overexpressing plasmid for pleu-MU402 was constructed by using the expression vector pMAT1552, which contains the *pyrG* gene of *M. circinelloides* to encode orotidine 5′-phosphate decarboxylase to produce uridine as a selectable marker. It is surrounded up-stream and down-stream by 1 kb of *carB-carRP* sequences to allow its chromosomal integration by homologous recombination. PCR amplification was carried out to isolate the *D15D* gene from the genome of *M. alpina* ATCC 32222 using the primers D15D-F/R (Table S1). The 30 bp homologous sequences of both sides of *XhoI* restriction site in pMAT1552 were also included in these primers. After digestion with *XhoI* restriction endonuclease, the PCR fragment was ligated to generate plasmid pMAT1552-D15D (Figure 5) for mutant pleu-MU402. The recombinant plasmid was transformed into *E. coli* Top 10 competent cells for its propagation. The extracted plasmids from these bacteria were checked by PCR analysis and DNA sequencing was carried out to confirm the gene sequence.

### 4.3. Gene Expression and RT-qPCR Analysis

*M. circenioides* was cultivated in a 2-L fermenter using K&R medium and mycelium was collected at 3, 24, 48, and 72 h. Total RNA of *M. circinelloides* was extracted using Trizol after grinding mycelia under liquid N_2_ and reverse-transcribed by using the Prime ScriptRT reagent kit (Takara) as described by manufacturer’s instructions. LightCycler 96 Instrument (Roche Diagnostics GmbH, Switzerland) with FastStart Universal SYBR Green Master (ROX) Supermix (Roche) was used to perform RT-qPCR by using primers D15DqPCR-F/R (Table S1). The expression level of mRNA was standardized to levels of 18S rRNA mRNA, and the results were analyzed as relative expression levels. The method of 2^−ΔΔC^ was used to quantify the obtained data.

### 4.4. Calculation of Glucose and Nitrogen Concentration from Culture Medium

The concentration glucose in the culture medium was measured by a glucose oxidase Perid-test kit according to the protocol supplied by the manufacturer (Shanghai Rongsheng Biotech Co., Ltd.). Ammonium concentration was determined by the indophenol method [41].

### 4.5. Determination of Cell Dry Weight, Total Fatty Acid and Fatty Acid Analysis

Biomass samples were collected on a weighed filter paper by filtration through a Buchner funnel under reduced pressure. These were washed three times with distilled water, frozen at −80 °C overnight and then dried by freeze dryer. The weight of the empty fatty acid extraction tube was taken. The extraction of fatty acids was carried out using 20 mg dry mycelia. Chloroform/methanol (2:1, *v*/*v*) was used to extract total fatty acids. Finally, the final weight of tube with fatty acids was taken. Total fatty acid was determined using following equation:

(1) $$Total\;FA=\frac{T1-T0}{\mathrm{Wm}}\times 100$$

Here, T_1_ = weight of tube with fatty acid; T_0_ = weight of empty tube; Wm = weight of mycelia.

For fatty acid analysis 10 mg of dry mycellia was taken. Fatty acid was extracted using the same method and methylated with 10% (*v*/*v*) methanolic HCl at 60 °C for 3 h. Pentadecanoic acid (15:0) (Sigma-Aldrich) was added into the freeze-dried cells as an internal standard and n-hexane were used to extract the fatty acid methyl esters, which were analyzed by GCFID equipped with a 30 m × 0.32 mm DB-Waxetr column with 0.25 μm film thickness. The program was as follows: 120 °C for 3 min, ramp to 200 °C at 5 °C per min, ramp to 220 °C at 4 °C per min, hold 2 min. 

### 4.6. Statistical Analysis

SPSS 16.0 for Windows (SPSS Inc. Chicago, IL) software was used to complete the statistical analysis. Three independent experiments were carried out and the data obtained from these experiments were used to calculate the mean values and standard error of the mean. The differences between means of the test were calculated by Student’s *t* test, and *p* < 0.05 was considered as significantly different.

### 4.7. Ethics Approval and Consent to Participate

Any experiments with human or animal participants were not performed by any of the authors.

## 5. Conclusions

This study constructed a SDA-producing *M. circinelloides* CBS 277.49 by *D15D* gene overexpression. According to our knowledge, this is the first study about the construction of a SDA-producing strain by gene cloning and recombination in *Mucor*. This work establishes a new scope for further research for improved production of SDA in *M. circinelloides*, specifically in its high lipid-producing strain, WJ11.

## Figures and Tables

**Table 1:** The fatty acid composition in *D15D* overexpressing strains.

Time	Fatty Acid Composition (relative %, *w*/*w*)						
Hour	C(16:0)	C(18:0)	C(18:1) OA %	C(18:2) LA %	C(18:3) GLA %	C(18:3) ALA %	C(18:4) SDA %
**Mc-1552**							
12 h	-	-	32.38 ± 1.33	30.87 ± 0.96	36.75 ± 0.88	-	-
24 h	29.10 ± 0.75	7.84 ± 0.58	20.43 ± 0.44	16.50 ± 0.67	26.13 ± 0.37	-	-
36 h	29.91 ± 0.52	7.60 ± 0.27	22.57 ± 0.11	14.39 ± 0.38	25.53 ± 0.23	-	-
48 h	22.23 ± 0.64	4.32 ± 0.44	26.68 ± 0.32	16.56 ± 0.25	30.21 ± 0.10	-	-
60 h	24.72 ± 0.07	3.70 ± 0.15	25.85 ± 0.08	15.78 ± 0.05	29.95 ± 0.05	-	-
72 h	24.92 ± 0.25	2.71 ± 0.32	26.09 ± 0.22	15.68 ± 0.30	30.60 ± 0.20	-	-
**Mc-D15D**							
12 h	37.23 ± 1.68	14.89 ± 1.93	16.24 ± 1.3	16.91 ± 1.13	14.73 ± 0.95	-	-
24 h	28.47 ± 0.44	9.08 ± 1.96	22.15 ± 0.77	18.91 ± 0.99	15.05 ± 0.58	3.73 ± 0.44	2.62 ± 0.38
36 h	17.79 ± 1.73	5.87 ± 0.68	26.99 ± 1.28	18.60 ± 0.97	21.41 ± 1.22	4.38 ± 1.1	4.97 ± 0.96
48 h	13.74 ± 0.09	2.96 ± 0.87	30.08 ± 0.03	17.61 ± 0.29	27.50 ± 0.06	3.13 ± 0.05	5.09 ± 0.08
60 h	12.60 ± 0.68	2.37 ± 0.28	32.76 ± 0.47	16.41 ± 0.49	28.55 ± 0.53	2.60 ± 0.47	4.70 ± 0.17
72 h	12.65 ± 0.07	2.03 ± 0.55	34.26 ± 0.08	15.90 ± 0.59	28.84 ± 0.44	2.13 ± 0.37	4.20 ± 0.28

## Supplementary Materials

The following are available online at https://www.mdpi.com/1422-0067/20/7/1683/s1, Table S1. The sequences of primers used in this work.

## Abbreviations

SDA	Stearidonic acid
LA	Linoleic acid
ALA	Alpha linolenic acid
GLA	Gamma linolenic acid
ARA	Arachidonic acid
EPA	Eicosapentaenoic acid
DHA	Docosahexaenoic acid
PUFA	Polyunsaturated fatty acid
GC	Gas chromatography
VLCPUFA	Very-long-chain polyunsaturated fatty acid
CDW	Cell dry weight
SAFA	Saturated fatty acids
MUFA	Mono-unsaturated fatty acids
TFA	Total fatty acids
