# Supplementary material for: Genetic Modification of Mucor circinelloides to Construct Stearidonic Acid Producing Cell Factory
Source: Int J Mol Sci. 2019 Apr 4;20(7):1683. doi: 10.3390/ijms20071683 (PMC6480972; doi:10.3390/ijms20071683)
Supplement: Supplementary file 1 [file ijms-20-01683-s001.pdf]

Table S1. The sequences of primers used in this work.

Primer	Sequence (5′ to 3′)[a]	Restriction enzyme	Application
D15D-F:	ACTTTTATATACAAAATAACTAAATCTCGAGATGGCACCCCCTCACGTTG	XhoI	Gene cloning
D15D-R	ACTAGTCGCAATTGCCGCGGCTCGAGCTAATGCTTGTAGAACACTACG	XhoI	Gene cloning
D15DqPCR-F	CTCGATGGTCTTCTCGCCCTTG		RT-PCR analysis
D15DqPCR-R	CACCGCGCTGGAAGTTCC		RT-PCR analysis
1552-F	CCTCGGCGTCATGATGTTTTTGTGTACCT		Gene checking
1552-R	GGGATGTCTGCTGCTACCATGTCTCAT		Gene checking

[a] Restriction enzyme sites are underlined.
